# Purification and characterization of a novel lipopeptide from *Streptomyces amritsarensis* sp. nov. active against methicillin-resistant *Staphylococcus aureus*

**DOI:** 10.1186/s13568-014-0050-y

**Published:** 2014-06-28

**Authors:** Deepika Sharma, Santi M Mandal, Rajesh Kumari Manhas

**Affiliations:** 1 Department of Microbiology, Guru Nanak Dev University, Amritsar 143005, Punjab, India; 2 Central Research Facility, Indian Institute of Technology, Kharagpur 721302, West Bengal, India

**Keywords:** Antibacterial, Novel, Lipopeptide, Thermostable, Non-toxic

## Abstract

Nowadays antimicrobial lipopeptides are being widely exploited for developing potential therapeutic agents for treating bacterial infections. In the present study, we have purified and characterized an antimicrobial lipopeptide produced by *Streptomyces amritsarensis* sp. nov. (= MTCC 11845^T^ = JCM 19660^T^). The lipopeptide was purified using silica gel chromatography, size exclusion chromatography and reverse phase- HPLC. The MS/MS analysis of the lipopeptide revealed that it has amino acid sequence as Ala-Thr-Gly-Ser-His-Gln and a long chain fatty acid tail with six times repeated the molecular mass of 161 Da which is corresponding to -C_12_H_19_. Based on the molecular mass (878.5 Da) and amino acid composition, the lipopeptide was identified as a novel lipopeptide. The MIC values of purified lipopeptide against *Bacillus subtilis* (MTCC 619)*, Staphylococcus epidermidis* (MTCC 435), *Mycobacterium smegmatis* (MTCC 6) and clinical strain, Methicillin Resistant *Staphylococcus aureus* (MRSA) were found to be 10, 15, 25 and 45 μg/ml, respectively. It was completely stable at 70°C for 1 h and retained 81.8% activity after autoclaving (121°C for 15 min). It did not show any change in its activity profile between pH 5.0 - 9.0 and is stable to trypsin, proteinase K and lipase enzymes. It was found to be non-mutagenic against *Salmonella typhimurium* (TA98) and did not show cytotoxicity when checked against Chinese hamster ovary (CHO) cell line. In addition to antibacterial activity it also exhibits biosurfactant activity.

## Introduction

In the last two decades, exploring the possibility of developing new classes of antimicrobial compounds has emerged prominently due to pathogens acquired resistance to emerging antibiotics. Presently available antimicrobial compounds are getting old and less efficient, only few truly original replacements are available. This is due to a number of different reasons including daunting R&D costs for putting a new molecule on a highly competitive market and the inherent difficulty of identifying innovative antibiotic targets (Nathan and Goldberg [2005]; Payne et al. [2007]). Therefore, screening and characterization of the novel antimicrobial compounds especially, peptides from microorganisms, have drawn attention (Pirri et al. [2009]; Laverty et al. [2011]). Among antimicrobial peptides, lipopeptides are small molecules and have been considered as potential source of future antibiotics because of their different mechanisms of action as compared to conventional antibiotics (Baindara et al. [2013]).

Antimicrobial lipopeptides represent an old class of antibiotics that were discovered over 50 years ago and consist of a hydrophilic cyclic peptide portion attached to a fatty acid chain. They are biosynthesized by large multi-enzymes called non-ribosomal peptide synthetases *via* non-ribosomal pathways (Pirri et al. [2009]). All antibiotics belonging to this novel class contain multiple nonproteinogenic amino acids as well as different lipid tails; this yields remarkable structural diversity (Strieker and Marahiel [2009]). Lipopeptides easily bind to the bacterial surface bilayer and alter the local lipid organizational linking on negatively charged fatty acids resulting in restructuring of the lipid bilayer and prevent cellular processes (Horn et al. [2012]; Mandal et al. [2013]).

Actinobacteria especially, species of the genus *Streptomyces* are reported to produce diverse antimicrobial lipopeptides with their applications in pharmaceutical industries. Amphomycin was the first lipopeptide to be discovered from *Actinoplanes friuliensis*, followed by a number of related antibiotics, including crystallomycin, aspartocin, glumamycin, laspartomycin, tsushimycin, and the best studied so far, daptomycin (Schneider et al. [2009]). As apparently 99% of the microbial species are still unexplored (Davies [1999]; Watve et al. [2001]) therefore, possibility of discovering actinobacteria producing potent and novel lipopeptides still remains. In the light of this, present study was aimed at purification and characterization of a novel antimicrobial lipopeptide produced by *Streptomyces amritsarensis*.

## Materials and methods

### Microorganism and maintenance

*Streptomyces amritsarensis* (= MTCC 11845^T^ = JCM 19660^T^) was isolated from soil and identified using polyphasic taxonomic approach (Sharma et al. [2014]). The culture was maintained on starch casein nitrate agar slopes at 4°C and as mycelial fragments and spores in 20% v/v glycerol at -80°C. All the test organisms except clinical isolates were procured from Microbial Type Culture Collection and Gene Bank (MTCC), Institute of Microbial Technology (IMTECH), Chandigarh, India. Clinical isolates *viz*. multi-drug resistant *E. coli*, MRSA and VRE were procured from local hospitals. They were maintained on nutrient agar slopes at 4°C.

### Growth and lipopeptide production profile

*S. amritsarensis* was grown in Starch Casein Nitrate (SCN) broth at 28°C with continuous shaking at 180 rpm for 10 days. Cell free supernatants were collected at one day intervals by centrifuging culture broths at 10,000 rpm for 30 min at 4°C and used for detection of lipopeptide antimicrobial activity using Kirby-Bauer disk diffusion susceptibility test (Bauer et al. [1966]). Filter paper discs (6 mm) impregnated with 10 μl of supernatants (filtered through 0.22 μm filter, Pall Lifesciences) were placed on Mueller-Hinton agar plate seeded with *Bacillus subtilis* (MTCC 619) and incubated at 37°C for 24 h. For the determination of growth, absorbance of culture broths were read at 600 nm and growth curve was prepared. Protein contents of culture supernatants were determined using Lowry method (Lowry et al. [1951]).

For large scale production of lipopeptide batch fermentation of *Streptomyces* strain was carried out in SCN broth at 28°C on a rotary shaker at 180 rpm. The flasks were inoculated with 2% by volume of seed culture, grown at 28°C for two days. Fermentation was terminated on 4^th^ day, at which the maximum production was obtained. The active compound from cell free supernatant was adsorbed on resin XAD-4 (5%) at 15°C under shaking conditions for 2 days and recovered from resin by eluting with methanol. The methanol fraction was dried using a rotavapour (BUCHI Rota vapor R-200) and dried residue was re-dissolved in small volume of methanol.

### Purification of lipopeptide

For the purification of the lipopeptide silica gel column chromatography (60-120 mesh) was carried out. Column (35 × 1.0 cm) was packed with silica gel using chloroform as solvent. The methanol extract was loaded at the top of the column and eluted step-wise with 100% CHCl_3_, 95:5, 90:10, 75:25, 70:30, 50:50 (v/v) of CHCl_3_: CH_3_OH solvent and 100% CH_3_OH (200 ml each) at a flow rate of 2 ml/min. Fractions were concentrated and redissolved in the same solvent ratio from which they were recovered. Fractions showing antimicrobial activity were pooled and further purified by subjecting to size exclusion chromatography using Toyopearl resin HW-40 and methanol as an eluent. The methanol fractions as such were subjected to antimicrobial activity, active fractions were combined and solvent was evaporated using a rota vapour, the residue was re-dissolved in Milli-Q water. Further purification was achieved through reverse phase HPLC (1260 Infinity, Agilent Technologies, USA) using a semi-preparative C18 column (Pursuit 10C18 250 × 21.2 mm) and acetonitrile: water (5:5) as mobile phase. Collected fractions were concentrated by speed vacuum and screened for antimicrobial activity. Lipopeptide was purified to homogeneity using reverse phase- HPLC (Agilent 1100 series) with a ZORBAX 300-SB18 column (4.6 mm × 250 mm, particle size 5 μm), at a flow rate of 1 ml/min. The solvent system used was 0.1% aqueous TFA (A) and acetonitrile containing 0.1% TFA (B). The gradient of solvent B used to run the column was as follows: 0-60% for 0–45 min, 60-80% for 45–55 min and 80-100% for 55–60 min. The elution from the column was monitored at 215 nm in a diode array detector and all the peaks of HPLC chromatogram were collected using a fraction collector (GILSON, France) coupled with the system. Collected fractions were concentrated by speed vacuum and screened for antimicrobial activity.

### MALDI-TOF-MS and sequencing of lipopeptide

Lipopeptide was characterized using Matrix-assisted laser desorption ionization (MALDI). The purified lipopeptide was lyophilized and re-suspended in methanol. Solution (4 μl) was mixed with 4 μl of matrix (CHCA, 10 mg/ml), 1.0 μl of this mixture solution was spotted onto the MALDI 100 well stainless steel sample plate and allowed to air dry prior to the MALDI analysis (Mandal et al. [2009]). MALDI mass spectra was obtained using a Voyager time-of-flight mass spectrometer (Applied Biosystem, USA), equipped with 337 nm N2 laser and operated in accelerating voltage 20 kV. The spectra were recorded in positive ion linear mode. To check the reproducibility of the spectrum, sample was separately spotted several times.

For peptide MS/MS sequencing, lipopeptide was incubated with 10% NaOH in methanol at room temperature for 16 h to cleave the lactone ring. The cleaved lipopeptide was lyophilized, again extracted with methanol and allowed for mass spectrometry analysis. The spectra were recorded in the post-source decay (PSD) ion mode as an average of 100 laser shots with a grid voltage of 75%. The reflector voltage was reduced in 25% steps and guide wire was reduced 0.02–0.01% with an extraction delay time of 100 ns.

### Fatty acid analysis of lipopeptide by GC-MS

Lipopeptide was subjected to acid hydrolysis by incubating the lipopeptide (5 mg) with 0.5 ml of 6 M HCl at 90°C for 18 h in sealed tubes. The fatty acids were extracted with ether and esterified with 0.95 ml methanol and 0.05 ml of 98% H_2_SO_4_ at 65°C for 6 h. Fatty acid methyl esters were obtained after extraction with n-hexane and analyzed by GC-MS with a Clarus 500 GC (PerkinElmer, USA). Helium was used as carrier gas at a flow rate of 1.0 ml/min. The column temperature was maintained at 120°C for 3 min and thereafter gradually increased (8°C/min) to 260°C.

### Determination of antimicrobial activity

Sensitivity of test organisms to purified lipopeptide was measured in terms of zone of inhibition using Kirby-Bauer disk diffusion susceptibility test (Bauer et al. [1966]). The plates containing Mueller-Hinton agar, yeast malt agar and potato dextrose agar were seeded with test bacteria, yeasts and fungi, respectively. Filter paper discs (6 mm) impregnated with 10 μg of lipopeptide were placed on media plates. The diameter of the resultant zone of inhibition was measured in mm after 24- 48 hours of incubation. Each experiment was performed in duplicates and repeated thrice. Various test organisms used in the study included *Bacillus subtilis* (MTCC 619), *Mycobacterium smegmatis* (MTCC 6), *Staphylococcus epidermidis* (MTCC 435), *Escherichia coli* (MTCC 1885), *Klebsiella pneumoniae* sub sp. *pneumoniae* (MTCC 109), *Enterobacter aerogenes* (MTCC 111), *Salmonella typhi* (MTCC 733), multi-drug resistant *E. coli*, MRSA, VRE, *Candida albicans* (MTCC 3017), *Rhodotorula rubra* (MTCC 248), *Colletotrichum acutatum* (MTCC 1037), *Cercospora beticola* (GenBank acc. no. KJ461435), *Fusarium oxysporum* f.sp. *dianthi* (MTCC 6659), and *Alternaria brassicicola* (MTCC 2102).

### Determination of minimum inhibitory concentration

The MIC of purified lipopeptide was evaluated by using a microtiter plate dilution assay. Test bacteria were grown to logarithmic phase under optimal conditions (up to 0.3 OD) and the test was performed in triplicates. To each well of the microtiter plate, 200 μl of fresh nutrient medium and 50 μl of bacterial suspension were added. Subsequently, different dilutions (50 μl) of freshly prepared samples were added to each well. The first column of the microtiter plate was left as a blank, containing fresh medium only. The microtiter plates were incubated at 37°C and OD was measured at 600 nm at 24 and 48 h using ELISA microplate reader (Bio-rad, Model 680XR). The lowest concentration that inhibited growth of the test strain and did not show any increase in absorption after 48 h was considered as MIC of the lipopeptide for that strain.

### Effect of pH, temperature and enzymes on lipopeptide activity

Sensitivity to temperature was determined by incubating the purified lipopeptide at different temperatures *viz.* 50°C, 60°C, 70°C, 80°C , 90°C, 100°C and 121°C for different durations. To determine the optimum pH for activity, purified compound was incubated over pH range of 3.0–12.0 for 30 min at 37°C. The sensitivity of the lipopeptide (1.0 mg/ml) to enzymes was tested against proteinase K (~30 U/mg), trypsin (~10,000 U/mg) and lipase (~9 U/mg). All enzymes were purchased from Sigma Aldrich. Enzyme solutions were prepared at 1.0 mg/ml in 50 mM phosphate buffer (pH 7.0). Equal volumes of enzyme solution and antimicrobial compound (20 μl each) were mixed and incubated at 37°C for 12 h. The enzyme reaction was terminated by heating reaction mixture at 80°C and residual activity was determined by disc diffusion method.

### Safety evaluation of lipopeptide

Mutagenicity of the lipopeptide was evaluated by Ames test (Maron and Ames [1983]). This *Salmonella* reverse mutation test is based on histidine dependence and mutations in *Salmonella typhimurium* (TA98/ MTCC 1251, IMTECH, Chandigarh). Concentrations of the lipopeptide used for checking toxicity were 50 and 100 μg 0.1 ml^−1^ plate^−1^. The overnight grown, 0.1 ml bacterial culture and 0.1 ml of lipopeptide were added to 2.0 ml of top agar. The contents were mixed and poured onto glucose minimal agar plates immediately. The plates were inoculated and incubated at 37°C for 48 h. The experiment was repeated to confirm the results. To determine the spontaneous reversion which is characteristic of the tester strain (TA 98), negative control (0.1 ml bacterial culture + 0.1 ml DMSO plate^−1^) was run while 4-Nitro-o-phenylenediamine (20 μg 0.1 ml^−1^ plate^−1^) was used as a positive control mutagen. The mutagenic potential of the lipopeptide was determined by comparing the number of colonies with control plates where no test compound was added.

In vitro cytotoxicity was evaluated using sulforhodamine B dye assay (Skehan et al. [1990]). The Chinese hamster ovary cell line (CHO) was used for the assay. Camptothecin (CPT), an anticancer drug was used as standard. The 96-well tissue culture plate, containing different concentrations of lipopeptide (2.5- 25 μg/ml) and CHO cells, was incubated for 48 h and the cell growth was stopped with trichloroacetic acid (50% TCA, 50 μl). The optical density (OD) was recorded at 540 nm on ELISA reader and percent growth inhibition was calculated.

### Biosurfactant property of lipopeptide

Biosurfactant property of lipopeptide was determined using the qualitative drop-collapse test (Youssef et al. [2004]). In this method, mineral oil (2 μl) was added to 96-well microtitre plate. The plate was equilibrated for 1 h at 37°C and 5 μl of the lipopeptide (10 μg, dissolved in water) was added to the surface of the oil and drop shape was observed after 1 min. The surface tension of a lipopeptide (0.2%, w/v) was measured using Du-Nouy-Ring method (Du Noüy and Pierre [1925]).

## Results

### Production and purification of lipopeptide

**Figure 1:**
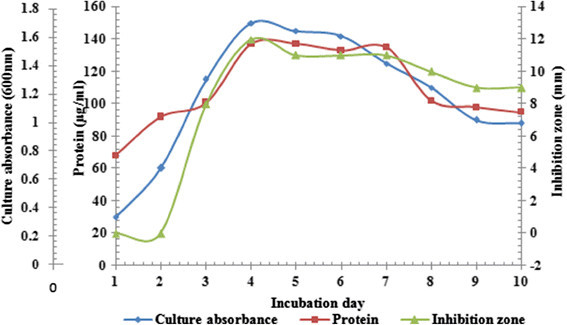
**Growth and lipopeptide production profile of** ***S. amritsarensis.***

Lipopeptide production and growth profile of *S. amritsarensis* is shown in Figure 1. Antimicrobial activity appeared in culture supernatant during late logarithmic phase. A significant increase in production (as measured by inhibition zone) and growth were observed with further incubation. The maximum growth and production were attained after 4 days which remained constant for 7 days and declined slightly with further incubation.

**Figure 2:**
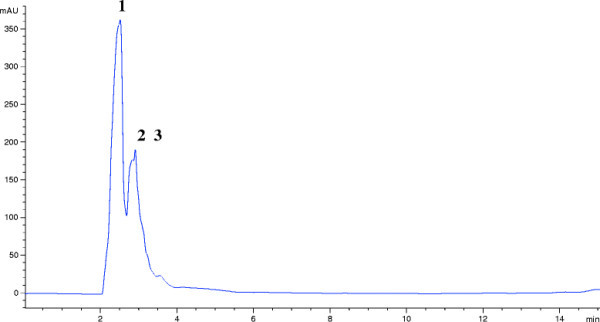
**Elution profile of lipopeptide using HPLC reverse phase chromatography on ZORBAX 300-SB18 column monitoring by absorbance at 215 nm.** Peak 3 corresponds to lipopeptide elution.

For purification of compound, production was carried out in SCN broth for 4 days. The active compound from supernatant was adsorbed on resin XAD-4 and recovered by eluting with methanol. Antimicrobial compound was partially purified by silica gel chromatography and size exclusion chromatography using Toyopearl resin HW-40. Reverse phase- HPLC of the partially purified compound using a semi-preparative C18 column revealed the presence of five peaks (Additional file 1: Figure S1). After lyophilization, all collected peaks (fractions) were tested for antimicrobial activity. Peaks 1, 2 and 3, demonstrating antimicrobial activity, were further resolved using ZORBAX 300-SB18 column (Figure 2).

### Lipopeptide MS/MS sequencing

**Figure 3:**
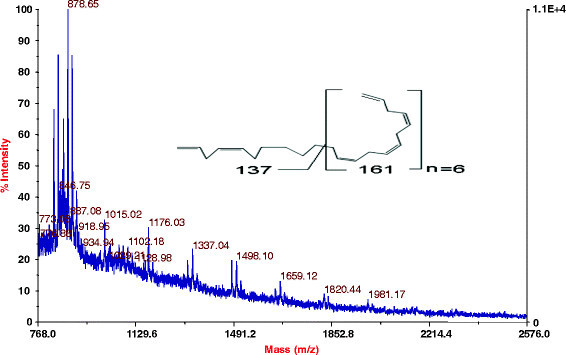
MALD-TOF mass spectrometry analysis of lipopeptide.

**Figure 4:**
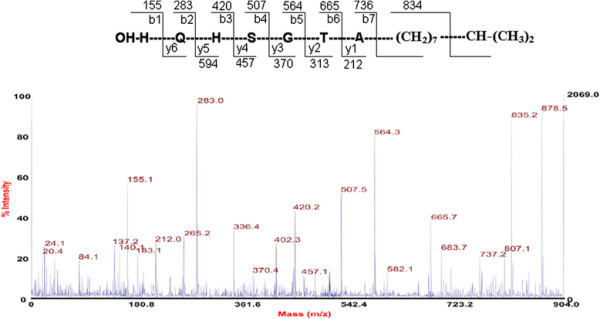
MALDI TOF PSD (MS/MS) spectrum of lipopeptide (Peak 3) and amino acid sequence of the lipopeptide obtained by de novo sequencing.

The primary structure of lipopeptide (peak 3) was elucidated using a combination of mass spectrometry techniques. The peaks obtained for different fragments at m/z 834, 736, 665, 564, 507, 420, 283 and 155 in MS/MS analysis revealed the lipopeptide sequence as Ala-Thr-Gly-Ser-His-Gln. The C-terminal amino acid in peptide is linked to aliphatic chain of -(CH_2_)_7_-CH_2_-(CH_3_)_2_- with a total mass value of m/z 878.5 Da (Figure 3). Further, MALDI TOF MS analysis clearly showed an addition of 137 Da mass unit corresponding to -C_10_H_17_- shown in figure inset (Figure 4). Interestingly, there is a long tail of fatty acid chain with six times repeated the molecular mass of 161 Da which is corresponding to -C_12_H_19_ (Figure 3). Based on the molecular mass and amino acid composition, the lipopeptide was identified as a novel lipopeptide.

### Inhibition spectrum and sensitivity of the lipopeptide

**Figure 5:**
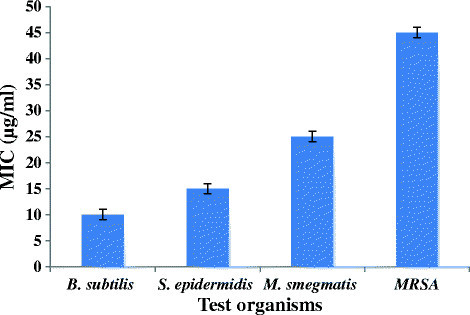
Determination of MIC for lipopeptide.

The purified lipopeptide (10 μg) showed activity only against Gram positive bacteria with inhibition zones of 21, 17, 15 and 13 mm against *B. subtilis*, *S. epidermidis, M. smegmatis* and MRSA, respectively (Additional file 1: Figure S2). It did not show activity against any of the tested Gram-negative bacteria and fungi. The MIC assay for test organisms with purified lipopeptide using micro-titer plates in triplicates revealed lowest MIC value of 10 μg/ml against *B. subtilis* and highest against MRSA i.e 45 μg/ml (Figure 5).

The results of heat stability assay demonstrated that the lipopeptide was completely stable at 70°C for 1 h and a loss of 13.7% was observed at 100°C after 15 minutes incubation. After autoclaving (121°C for 15 min) antimicrobial activity was reduced by 18.2%. It did not show any change in its activity profile between pH 5.0–9.0 (Table 1). Lipopeptide was found to be resistant to trypsin and lipase, and negligible loss in activity was observed after treatment with proteinase K.

**Table 1:** Factors affecting lipopeptide activity

**Treatment**	**Reaction duration/condition**	**Residual activity (%)**
pH		
2	1 h/4°C	0
3	1 h/4°C	72.7
4	1 h/4°C	84.6
5	1 h/4°C	100
6	1 h/4°C	100
7	1 h/4°C	100
8	1 h/4°C	100
9	1 h/4°C	100
10	1 h/4°C	86.3
11	1 h/4°C	68.1
12	1 h/4°C	0
Temperature (°C)		
50	1 h	100
60	1 h	100
70	1 h	100
80	1 h	84.2
90	15 min	95.0
100	15 min	86.3
121	15 min (304 KPa)	81.8

### Safety evaluation

Mutagenicity of lipopeptide was checked by Ames test at two concentrations *viz.* 50 and 100 μg/0.1 ml. The number of revertant colonies were counted after 48 hours of incubation at 37°C and compared with the negative control. The number of revertant colonies in the presence of lipopeptide was found to be same as in the negative control (21 ± 2.0) for TA98. However, the number of colonies in presence of positive mutagen (20 μg plate^-1^) was found to be considerably higher. The results of the present study indicate that the lipiopeptide is non-mutagenic.

**Figure 6:**
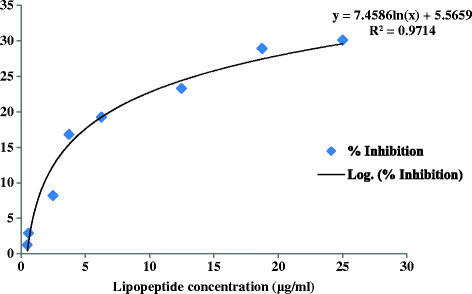
Cytotoxic effect of lipopeptide on CHO cell line.

**Figure 7:**
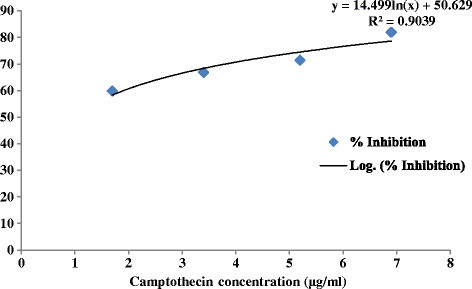
Cytotoxic effect of camptothecin on CHO cell line.

The effect of lipopeptide at different concentrations, 2.5–25 μg/ml on chinese hamster ovary (CHO) cell line is shown in Figure 6. It showed 2.9, 16.8, 19.25, 23.28 and 28.9% growth inhibition at concentrations of 2.5, 6.25, 12.5, 18.75 and 25 μg/ml, respectively. The IC_50_ value of lipopeptide was found to be 387 μg/ml which is very high as compared to the IC_50_ value of standard drug (0.959 μg/ml). The cytotoxicity % inhibition of standard (camptothecin) is shown in Figure 7. This indicates that it do not exhibit cytotoxicity.

### Lipopeptide as biosurfactant

The lipopeptide collapsed the oil drop and lowered surface tension of water from 72 to 37 mN m^−1^ when used at a concentration of 0.2%. These results show that it possesses good surfactant activity.

## Discussion

Due to few antibiotics and free availability of effective antibiotics against diverse bacterial species the antimicrobial era is threatened by high levels of antibiotic resistance (Song [2008]). Among infections caused by antibiotic resistant Gram-positive bacteria, MRSA and VRE are of particular concern (Rice [2008]) and this lead to the discovery of potential antibiotics such as vancomycin, teicoplanin, synercid (quinipristin and dalfopristin), tigecycline and linezolid. Daptomycin is the recent FDA-approved lipopeptide, exhibiting potent activity against a broad range of Gram-positive pathogens especially, MRSA and VRE. However, rare incidences of clinical resistance have also been reported against daptomycin (D’Costa et al. [2012]). Since resistance to each new antibiotic ultimately emerges, usually within few years after it is marketed, there is always a need to find new antimicrobial agents to combat antibiotic resistant strains of pathogenic and opportunistic pathogenic microorganisms.

Since the late 1960s, only two novel classes of antibiotics, the oxazolidinones and the cyclic lipopeptides, have entered the market (Rodriguez de Castro et al. [2009]). Thus antibiotic market is looking to increase the number of new products with improved effectiveness. Nowadays, a huge amount of resources is being invested in R&D to find novel antimicrobials that can solve the problem of antibiotic resistance (Maria-Neto et al. [2012]). The market for antibacterial drugs is highly competitive, and many companies are engaged in the development of anti-MRSA or multi-drug-resistant treatments, where lipopeptides are the target compounds of most companies (Mandal et al. [2013]). Antimicrobial lipopeptides are nonribosomally synthesized, having macrocyclic peptide cores consisting of eleven to thirteen amino acids, which are rigidified by the formation of a ten-membered ring. They are produced by NRPSs with variation of the fatty acid tail (Strieker and Marahiel [2009]). The mechanism of action of lipopeptides is distinct from those of other antibiotics currently on the market. They form pores in membranes of bacteria after oligomerization and these pores may cause transmembrane ion influxes, including Na + and K+, which result in membrane disruption and cell death. Two key properties of lipopeptides are: i) a number of lipopeptides tend to oligomerize and ii) their ability to interact with membranes via their lipid tail (Straus and Hancock [2006]). These unique properties of the lipopeptides prevent pathogens to develop resistance against them, thus makes them highly active against multidrug-resistant bacteria (Mangoni and Shai [2011]). Generally the bactericidal activity of the lipopeptide increases, with the addition of a lipid tail of appropriate length (typically C10–C12) and lipopeptides containing 14 or 16 carbon atoms in lipid tail length exhibit enhanced antifungal activity in addition to antibacterial activity (Mandal et al. [2013]). This can be due to either an increase in the affinity of the lipid tail for the hydrocarbon chains or as a result of the stronger interaction of the cationic peptide with the lipid headgroups (Straus and Hancock [2006]).

Antimicrobial lipopeptides have been purified from many bacterial genera including several *Streptomyces* spp. such as *S. violaceus* var. *aspartocinius* (aspartocin), *S. griseoflavus* (tsushimycin), *S. viridochromogens* (laspartomycin)*, S. coelicolor* (calcium-dependent antibiotic), *S. roseosporus* (daptomycin)*, S. fradiae* (A54145), *S. parvulus var. parvuli* (parvuline) and *S. canus* (amphomycin) (Schneider et al. [2009]). The present study reports the production of another novel lipopeptide from a *Streptomyces* sp. The primary structure of lipopeptide was determined using a combination of chemical reactions and mass spectrometry techniques. It consists of six amino acids linked to aliphatic chain of -(CH_2_)_7_-CH_2_-(CH_3_)_2_ and a long tail of fatty acid chain with six times repeated the molecular mass of 161 Da which is corresponding to -C_12_H_19_. Based on structure and molecular weight (878.5 Da), it is different from already reported lipopeptides *viz.* daptomycin (1620.6 Da), aspartocin (1317 Da), glumamycin (1290.4 Da), tsushimycin (1304.7 Da) and arylomycin A6 (867.4 Da). Lipopeptides vary in their amino acid and/or fatty acid composition and all these variations in length and branching of the fatty acid chains and amino acid substitutions lead to remarkable lipopeptide diversity and activities. Generally, they are reported to be thermostable, resistant to proteolytic enzymes and inhibit the growth of microorganism by altering the membrane integrity (Mandal et al. [2013]). Similarly, lipopeptide in the present study is found to be completely stable for 1 h at 70°C, retaining 81.8% activity even after autoclaving (121°C for 15 min). It exhibits absolute activity over a broad pH range of 5.0 –9.0 and found to be resistant to hydrolytic enzymes: trypsin, proteinase K and lipase. Similarly, paenibacterin, an antimicrobial lipopeptide produced by *Paneibacillus* sp. strain is reported to be resistant to trypsin and lipase enzymes (Guo et al. [2012]). The strong antibacterial activity of the present lipopeptide can be related to the carbon chain length (C12) of the lipid tail as bactericidal activity of the lipopeptides depends upon the length of the lipid tail. Safety evaluation of the lipopeptide demonstrates its non-cytotoxic and non-mutagenic nature which is a prerequisite for development of a drug.

In conclusion, *Streptomyces amritsarensis* produces a novel antimicrobial lipopeptide that is active against a variety of Gram-positive bacteria especially, MRSA. Its stability and non-toxic nature suggest that it may serve as a new pharmacological drug and an addition to the panoply of lipopeptide group of antibiotics. It may also be used in the cosmetics industry for developing skin-care products and shampoos as it possesses good surface active property and as emulsifier and bio-preservative in the food industry. Further antimicrobial spectrum of the lipopeptide can be enhanced by chemical modifications in the lipid tail length, increasing number of carbon atoms.

## Competing interests

The author(s) declare that they have no competing interests.

## Authors’ contributions

RKM: Conceived the study, designed and performed experiments, critically analyzed data and drafted manuscript. DS: Designed and performed all the experiments, analyzed data and drafted manuscript. SMS: Performed MALDI-TOF-MS and sequencing, elucidated lipopeptide structure and helped to draft manuscript. All authors have read and approved the final manuscript.

## Additional file

## Supplementary Material

Additional file 1: Figure S1. Ellution profile of the partially purified compound using HPLC reverse phase chromatography on C18 column monitoring by absorbance at 280 nm. **Figure S2.** Antibacterial activity of lipopeptide against: **(a)***B. subtilis***(b)***S. epidermidis***(c)***M. smegmatis***(d)** MRSA. Click here for file
