# Supplementary material for: Purification and characterization of a novel lipopeptide from Streptomyces amritsarensis sp. nov. active against methicillin-resistant Staphylococcus aureus
Source: AMB Express. 2014 Jun 28;4:50. doi: 10.1186/s13568-014-0050-y (PMC4077001; doi:10.1186/s13568-014-0050-y)
Supplement: Additional file 1: Figure S1. — Ellution profile of the partially purified compound using HPLC reverse phase chromatography on C18 column monitoring by absorbance at 280 nm. Figure S2. Antibacterial activity of lipopeptide against: (a)B. subtilis(b)S. epidermidis(c)M. smegmatis(d) MRSA. [file s13568-014-0050-y-S1.pdf]

**Title: Purification and characterization of a novel lipopeptide from *Streptomyces amritsarensis* sp. nov. active against methicillin-resistant *Staphylococcus aureus***

**Journal: AMB Express**

**Authors: Deepika Sharma[1] Santi M Mandal[2] and Rajesh Kumari Manhas[1*]**

[1]Department of Microbiology, Guru Nanak Dev University, Amritsar, Punjab, 143005, India

[2]Central Research Facility, Indian Institute of Technology, Kharagpur, West Bengal 721302, India.

***Corresponding author:**

Dr. Rajesh Kumari Manhas

Associate Professor,

Department of Microbiology

Guru Nanak Dev University, Amritsar, Punjab, 143005, India

Email: rkmanhas@rediffmail.com

Tel: +91-183-2258802-09 Ext.3450; Fax: +91-183-2258819-20.

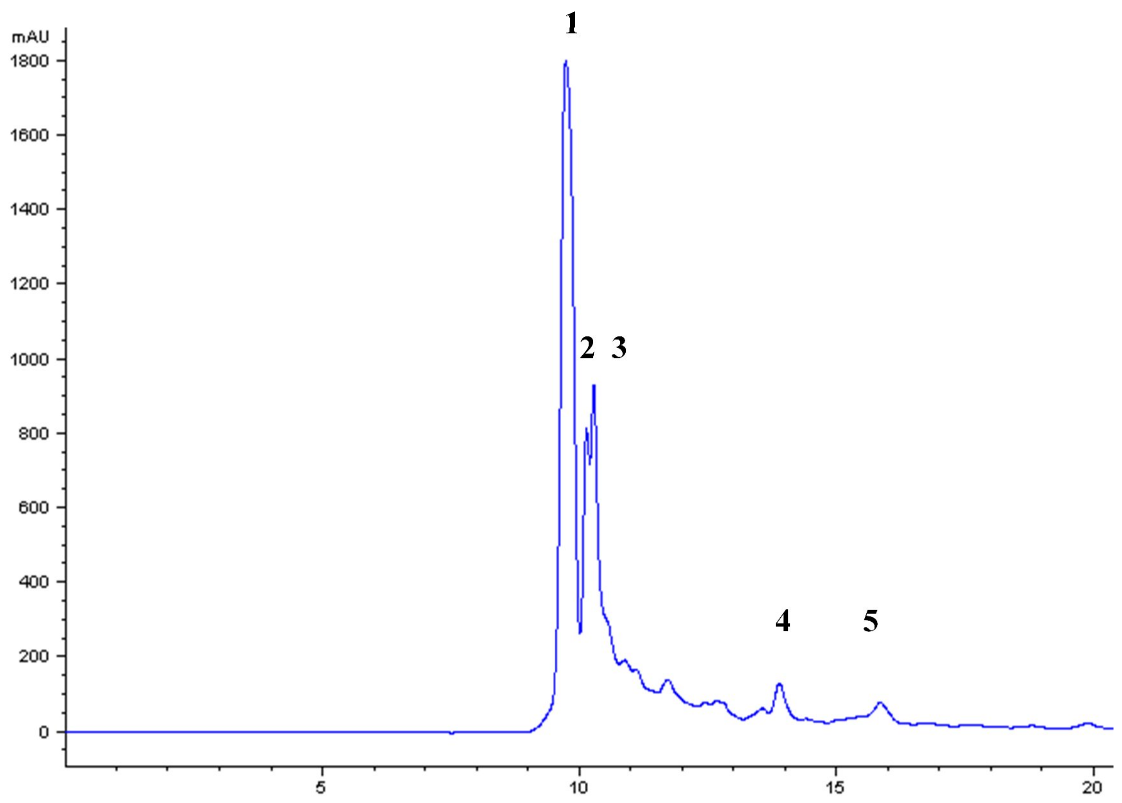


**Fig. S1 Ellution profile of the partially purified compound using a HPLC reverse phase chromatography on C18 column monitoring by absorbance at 280 nm**

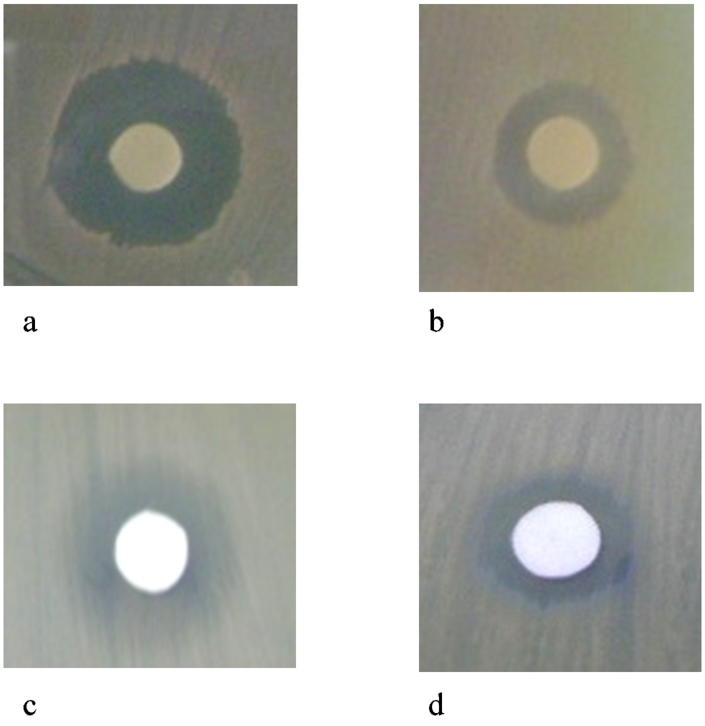

Fig. S2 Antibacterial activity of lipopeptide against: (a) ***B. subtilis,*** (b) ***S. epidermidis*** c) ***M. smegmatis*** (d) MRSA
